# CpG plus radiotherapy: a review of preclinical works leading to clinical trial

**DOI:** 10.3389/fonc.2012.00101

**Published:** 2012-08-14

**Authors:** Kathy A. Mason, Nancy R. Hunter

**Affiliations:** Department of Experimental Radiation Oncology, University of Texas MD Anderson Cancer CenterHouston, TX, USA

**Keywords:** CpG, oligodeoxynucleotides, radiotherapy, immunotherapy

## Abstract

Studies performed three decades ago in our laboratory supported the hypothesis that radiation efficacy may be augmented by bacterial extracts that stimulate non-specific systemic antitumor immune responses. Application to the clinic was halted by unacceptable side effects and toxicities resulting from exposure to whole bacterial pathogens. Later scientific advances demonstrated that DNA isolated from bacteria was immunostimulatory and could be reproduced with synthetic oligodeoxynucleotides (ODNs), thus fueling the transition from bugs to drugs. Unmethylated CpG motifs within bacterial DNA induce activation of Toll-like receptor 9 and subsequently activate antigen-specific cellular immune responses. CpG ODNs have demonstrated favorable toxicity profiles in phase I clinical trials. We showed that this potent immunoadjuvant can be used in combination with radiation therapy to enhance local and systemic responses of several murine tumors. Studies demonstrated that enhanced tumor response is mediated in part by the host immune system. Antitumor efficacy was diminished in immunocompromised mice. Animals cured by combination of radiation and CpG ODN were resistant to subsequent tumor rechallenge. This body of work contributes to our understanding of the dynamic interplay between tumor irradiation and the host immune system and may facilitate translation to clinical trials.

## INTRODUCTION

The immune system can influence growth of malignant tumors and responses to therapy with radiation or cytotoxic drugs. Immune deficiency can lower tumor response to conventional treatments, whereas stimulation of the immune system may enhance therapeutic responses (Dunn et al., 2002). This understanding led to the use of immunologic approaches for cancer treatments as monotherapy or in combination with chemotherapy or radiotherapy. In early developmental stages of cancer immunotherapy, bacteria or bacterial extracts, such as *Bacillus Calmette–Guérin* and *Corynebacterium parvum* were used to stimulate antitumor immunity (Yron et al., 1973; Milas and Scott, 1978). These bacteria or their extracts elicited or augmented many facets of immunological reactions, including macrophage and natural killer cell activation, induction of antibody-dependent cell cytotoxicity, and production of cytokines with antitumor activity. They were shown to be potent antitumor agents in a variety of rodent tumors, and they improved the efficacy of chemotherapy and radiotherapy (Yron et al., 1973; Milas and Scott, 1978). In contrast with promising preclinical results, however, these first-generation bacterial immunotherapeutics provided only modest clinical benefits (Mihich and Fefer, 1983). In addition, patients given multiple treatments of whole bacteria and their crude extracts showed symptoms of toxicity, including fever, nausea, vomiting, and pain at the injection site (Milas and Scott, 1978; Mihich and Fefer, 1983).

Recent advances in immunotherapy led to the discovery that immunostimulatory activity of bacteria resides in their DNA (Tokunaga et al., 1999), notably in unmethylated CpG motifs (Krieg et al., 1995) prevalent in bacterial but not in vertebrate genomic DNA. This led to chemical synthesis of oligodeoxynucleotides (ODNs) containing unmethylated CpG motifs that are recognized by immune cells expressing Toll-like receptor 9 (TLR9) in plasmacytoid dendritic cells and B cells (Hemmi et al., 2000). By stimulating TLR9, CpG ODNs induce a cascade of cellular and molecular responses leading to secretion of antigen-specific antibodies and cytokines and chemokines that trigger a wide range of secondary effects such as natural killer cell and monocyte activation (Uhlmann and Vollmer, 2003). Importantly, this receptor-mediated signaling pathway activates both innate and adaptive immunological reactions with less toxicity than do whole bacteria or their extracts (Hemmi et al., 2000). Early studies using CpG in experimental animals showed that these ODNs slowed tumor growth and prolonged tumor–host survival (Blazar et al., 2001; Kawarada et al., 2001; Heckelsmiller et al., 2002; Baines and Celis, 2003; Lonsdorf et al., 2003; Weigel et al., 2003; Krieg, 2004). In addition, CpG ODN treatment improved the outcome of surgery and chemotherapy (Weigel et al., 2003; Krieg, 2004). Our group pioneered work showing that this potent immunoadjuvant can be used in combination with radiation therapy to enhance local and systemic responses in murine tumors (Milas et al., 2004; Mason et al., 2005).

## EARLY STUDIES: COMBINATION OF CORYNEBACTERIA AND RADIOTHERAPY

Earliest studies with systemic injections (iv) of *Corynebacterium granulosum* or *C. parvum* in mice showed that these agents could induce complete regression of established s.c. immunogenic fibrosarcomas (Milas et al., 1974a,b). The response of individual tumors was extremely variable: some regressed permanently and others grew only slightly more slowly than controls. *C. parvum* and *C. granulosum* also reduced the number of metastatic lung tumor nodules when mice were treated within a few days of i.v. injection of fibrosarcoma cells, and many mice were cured of metastatic disease (Milas et al., 1974a; Milas and Scott, 1978).

These results led to studies to determine whether non-specific immunotherapy with *C. parvum* was an effective adjunct to radiotherapy, since treatment response depends not only on radiobiological factors but also on the immune response of the tumor-bearing host (Milas et al., 1975a; Milas and Scott, 1978; Milas, 1980). *C. parvum* increased radiosensitivity of well-established (8 mm diameter) immunogenic murine fibrosarcomas when local irradiation was given as a single-dose or in multiple fractions (Milas et al., 1975a,b; Milas, 1980). Combination treatment prolonged survival of mice more than radiotherapy or immunotherapy alone, and *C. parvum* significantly improved radiocurability. Tumors not cured by combination treatment grew more slowly and produced fewer metastases than tumors exposed to the individual treatments (Milas and Scott, 1978; Milas, 1980). In one study, local irradiation of a highly metastatic immunogenic mammary carcinoma with 60 Gy caused complete tumor regression but greatly increased the number of spontaneous lung metastases compared with mice whose primary tumors were surgically removed (Milas et al., 1976; Milas, 1980). *C. parvum* given before irradiation protected mice against this effect and reduced the frequency of lung metastases below that in mice whose tumor was surgically removed.

Therapeutic efficacy of immunotherapy plus radiotherapy was shown to depend on a number of factors including tumor size and immunogenicity, dose and route of *C. parvum* administration, and sequence of administration (Milas and Scott, 1978; Milas, 1980). Higher doses of local irradiation were required to cure immunogenic tumors in mice immunocompromised by whole-body irradiation (Stone and Milas, 1978; Milas, 1980), and *C. parvum* was less effective in augmenting radiocurability of weakly immunogenic tumors (Suit et al., 1976).

## BUGS TO DRUGS: CpG OLIGODEOXYNUCLEOTIDE AND RADIOTHERAPY

The discovery that immunostimulatory activity of bacteria resides in their DNA (Tokunaga et al., 1999), notably in unmethylated CpG motifs (Krieg et al., 1995), led to explorations of CpG ODN’s immunotherapeutic and immunomodulatory effects. Our recent studies demonstrated that synthetic CpG ODNs can be used as potent immunoadjuvants in combination with radiotherapy to enhance radioresponse of murine tumors (Milas et al., 2004; Mason et al., 2005). Experiments were performed using murine immunogenic fibrosarcomas growing in the leg of C3Hf/Kam mice. CpG ODN 1826 was administered one, three, or seven times s.c. peritumoral starting when tumors were 6 mm in diameter. CpG ODN 1826 monotherapy had minimal effect on tumor growth. Primary tumors were irradiated when they reached 8 mm in diameter. Response to radiotherapy was assessed by tumor growth delay and TCD50 (radiation dose yielding 50% tumor cures). The ODN dramatically enhanced tumor growth delay in response to single-dose irradiation by 2.58–2.65 and improved radiocurability, reducing TCD50 by a factor of 1.93, from 39.6 (36.1–43.1) Gy to 20.5 (14.3–25.7) Gy (Milas et al., 2004). Multiple administrations of the ODN were more effective than single administration. Importantly, improvement in radioresponse was also observed when CpG ODN 1826 was combined with conventional daily fractional doses of 2 Gy (Mason et al., 2005). A total dose of 83.1 (79.2–90.0) Gy was needed to achieve 50% tumor cure in mice treated with radiation plus the inactive ODN control and only 23.0 (11.5–32.7) Gy was needed when CpG ODN 1826 plus radiation was given. Tumor response to fractionated radiotherapy at the TCD50 level was potentiated by a radiation enhancement factor (EF) of 3.61, substantially higher than that observed for single-dose radiotherapy (EF 1.93). The superiority of CpG ODN treatment in combination with fractionated radiotherapy bodes well for translation of this treatment approach to the clinic.

**FIGURE 1:**
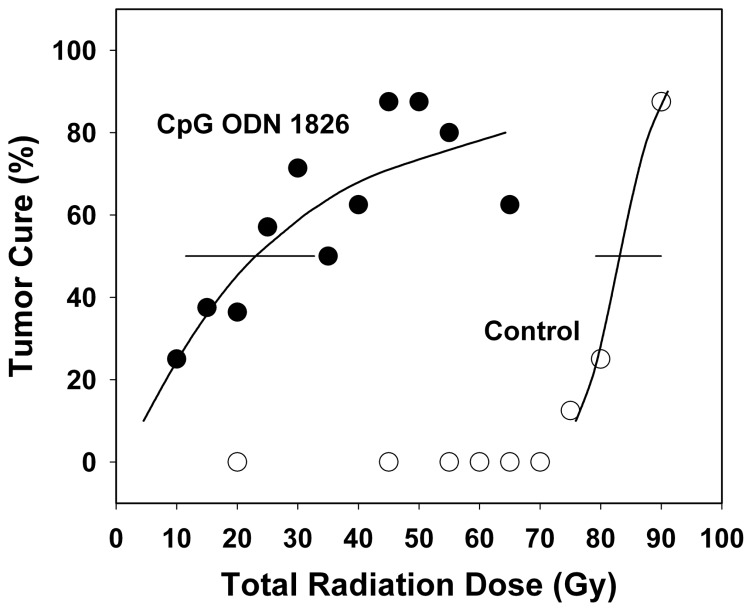
**Effect of CpG ODN 1826 on tumor radiocurability**. Percentage of tumor cures was plotted as a function of radiation dose. Mice bearing FSa tumors in the leg were exposed to a range of fractionated doses when tumors reached 8 mm in diameter and treated seven times with the active CpG ODN 1826 (●) or the inactive ODN 2138 (о), at a dose of 100 μg per mouse given s.c. peritumorally, when tumor diameters were 6 and 8 mm and once weekly for five additional weeks. The TCD50 was determined at 100 days after irradiation. *Horizontal bars*, 95% confidence intervals. Reprinted by permission from the American Association for Cancer Research (Mason et al., 2005).

Fractionated radiation cure probability curves are shown in **Figure 1**. The shallower slope of the CpG ODN 1826 plus radiation group most likely reflects heterogeneity of antitumor responses in mice treated with CpG ODN 1826. Variability in tumor response to combined treatment was also observed when tumor growth delay was the treatment endpoint. Since this fibrosarcoma grows rapidly, treatment with clinically relevant 2-Gy fractions twice a day for 5 days caused only a small delay in tumor growth. The effect of CpG ODN 1826 on radioresponse was initially observed several days after the start of irradiation in the fractionated protocol, when tumors had grown considerably. For example, some tumors began to regress after they grew as large as 9–14 mm, demonstrating that once elicited, the brisk antitumor response was capable of eliminating many cells in the large bulky tumors.

**FIGURE 2:**
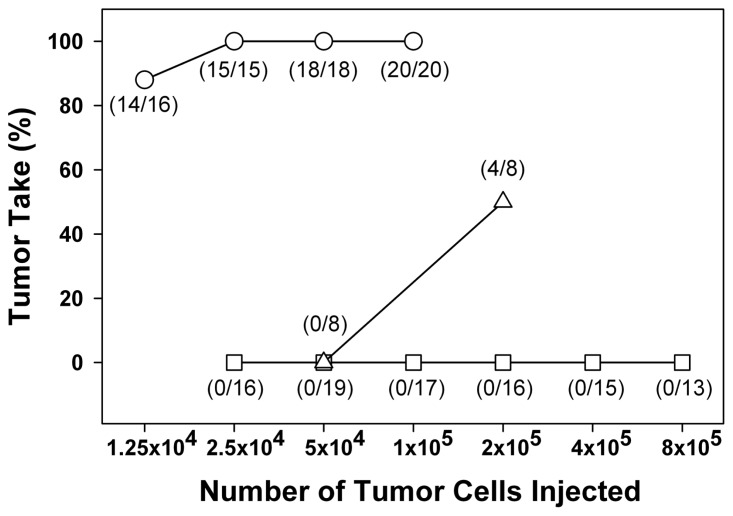
**Resistance of cured mice to reinoculation of tumor cells**. Mice cured of their primary tumor after irradiation alone (∆) or after treatment with CpG ODN 1826 plus irradiation (□) were reinoculated with FSa tumor cells 100–120 days after local tumor irradiation. Age-matched untreated mice were used as controls (о). Mice were injected s.c. on the abdomen with graded doses of FSa tumor cells and tumor takes observed for up to 2 months after inoculation. *Numbers in parentheses*, tumor takes over total injection sites. Reprinted by permission from the American Association for Cancer Research (Mason et al., 2005).

Mice cured of their fibrosarcoma by CpG ODN 1826 plus local irradiation were tested for resistance to tumor rechallenge (Mason et al., 2005). Mice cured of their tumor by treatment with either radiation alone or CpG ODN 1826 plus irradiation were resistant to subsequent s.c. tumor cell inoculation compared with previously untreated age-matched non-tumor-bearing mice (**Figure 2**). In normal mice, 100% tumor take was achieved with inoculations as low as 2.5 × 10^5^ tumor cells. At 100–120 days after treatment, mice cured by radiation alone required 2 × 10^5^ tumor cells to produce 50% tumor take, whereas mice treated with CpG ODN 1826 plus irradiation were totally resistant to tumor rechallenge with cell numbers as high as 8 × 10^5^. Like the animals rechallenged by the s.c. route, mice locally cured by CpG ODN 1826 plus irradiation were much more resistant to development of artificial metastases in the lung than were those cured by radiation alone. These results showed that the systemic antitumor rejection response generated by CpG ODN 1826 plus radiotherapy exerted antitumor effects long after exposure to the agents. Secondary tumor rejection was most likely due to development of a memory response and possibly specific T cell-mediated immunity (Koski and Czerniecki, 2005; Mason et al., 2005). A similar memory response was reported recently using a tumor vaccine composed of C-class CpG ODNs and irradiated melanoma tumor cells that induced long-term antitumor immunity against B16F1 tumors in mice (Cerkovnik et al., 2010).

The mechanisms of action of CpG ODNs for cancer immunotherapy have been reviewed in detail elsewhere (Krieg, 2001, 2006; Jahrsdorfer and Weiner, 2008; Krieg, 2008). We observed histological changes in fibrosarcomas treated with CpG ODN and radiation characterized by increased necrosis and heavy-infiltration of host inflammatory cells, primarily lymphocytes, and granulocytes (Milas et al., 2004). The specific nature of the antitumor rejection response at the primary tumor site and on metastases outside the irradiated field was subsequently investigated (Hart et al., 2008). An abscopal-like tumor model was used in which bilateral tumors in mice were left untreated in one hind leg and treated with radiation, CpG, or the combination in the contralateral leg. CpG ODN elevated systemic cytokine levels of IL-12p40, known to induce activation of NK cells and cytolytic CD8^+^ T cells, and IL-10, suggesting induction of antitumor antibody production. Compared to radiation alone, increased numbers of CD11c^+^ and CD8^+^ cytolytic T cells were found within the tumor draining lymph nodes following combined treatment with CpG ODN 1826 and local tumor irradiation. Enhanced local tumor control was accompanied by a measurable decrease in tumor burden at distant sites. A more recent study showed that fractionated (but not single-dose) radiotherapy induced an immune-mediated abscopal effect when combined with anti-CTLA-4 antibody in two preclinical rodent tumor models (Dewan et al., 2009).

Studies by other investigators suggested that CpG ODN induces antigen-specific antitumor T cell responses and activation of dendritic cells promoting strong immune memory responses (Shah et al., 2003). We hypothesized that when radiotherapy is given after CpG ODN injection, tumor antigens released from dying cells are taken up by activated dendritic cells, leading to induction of a tumor-specific T cell response. Others proposed that *in situ* tumor destruction by combination therapy may create a unique “*in situ* dendritic cell vaccine” (den Brok et al., 2006; Jahrsdorfer and Weiner, 2008). Radiotherapy has been reported to potentiate therapeutic efficacy of intratumoral dendritic cell vaccination (Teitz-Tennenbaum et al., 2008). Other possible mechanisms underlying the therapeutic efficacy of radiation with CpG ODNs include altered expression of critical molecules involved in immune recognition and killing by T cells; direct radiation damage to and killing of tumor cells, increased vulnerability of surviving cells to immune attack; or radiation-induced suppression of mechanisms inhibiting antitumor responses (Koski and Czerniecki, 2005). Subsequent investigations supported the theory that an immunoadjuvant effect of tumor cell death is an important aspect of radiotherapy response (Apetoh et al., 2007a). Radiation can promote changes in the tumor microenvironment that may enhance infiltration and activation of immune cells that have potential to influence tumor responses (Shiao and Coussens, 2010). Radiation was shown to up-regulate expression of CXL16 in tumors and to enhance recruitment and activation of CD8^+^ T cells (Matsumura et al., 2008; Matsumura and Demaria, 2010). Expression of MHC 1, important in antitumor T cell responses, was increased in a murine melanoma after irradiation (Lugade et al., 2005). Secretion of HMGB1 protein by lethally irradiated tumor cells and its effect on danger signaling was important in promoting antigen presentation (Apetoh et al., 2007b). Calreticulin exposure on the cell surface was shown to be required for the immunogenicity of radiation-induced apoptosis (Obeid et al., 2007; Formenti and Demaria, 2008).

Previously, we observed that enhancement of tumor radioresponse induced by CpG ODN 1826 was largely dependent on host immunocompetence (Milas et al., 2004). CpG ODN 1826 treatment of mice immunocompromised by sublethal whole-body irradiation caused only modest radiation-induced tumor growth delay of immunogenic fibrosarcoma, and the curative effect was lost. Since human tumors are generally considered to be weakly immunogenic, we tested the effect of CpG ODN 1826 on radioresponse of a non-immunogenic murine fibrosarcoma (Mason et al., 2005). CpG ODN enhanced radiation-induced tumor growth delay of non-immunogenic tumors when the ODN was injected s.c. (EF 1.41) or intratumorally (EF 1.73). Thus, in addition to being effective against the highly immunogenic fibrosarcoma, CpG ODN 1826 improved the radioresponse of a non-immunogenic tumor.

Several other animal tumor models have since shown response to combined therapy with CpG ODN and radiation. Treatment with CpG ODN and radiation-induced tumor remission in two-thirds of rats inoculated with 9L glioma (Meng et al., 2005). The combination treatment also enhanced tumor growth delay of s.c. B16F1 tumors (Cerkovnik et al., 2009). CpG ODN 1826 enhanced radiation-induced growth delay of Lewis lung cancer in mice and enhanced the apoptotic index in tumors given combined treatments compared to either treatment alone (Yuan et al., 2011). The combination of radiation with a CpG-based tumor vaccine significantly inhibited established LLC-OVA-carcinomas and cured about 60% of treated mice (Chamoto et al., 2009).

## CLINICAL TRIALS WITH CpG ODNs AND RADIOTHERAPY

Results with preclinical models suggested that CpG ODN would be more useful when combined with other therapeutic approaches in the treatment of cancer rather than as monotherapy (Krieg, 2006; Jahrsdorfer and Weiner, 2008). Although positive preclinical results are not necessarily predictive of clinical outcome, our findings provide compelling evidence that CpG ODN in combination with conventional radiotherapy is a strong candidate for clinical testing. Mice and humans have different TLR9 expression patterns, and so exposure to CpG motifs stimulates a narrower profile of cytokines/chemokines in humans than in mice (Krieg, 2008). Clinical trials are necessary to confirm the synergy between CpG ODNs and radiotherapy that was evident in preclinical testing.

Early clinical reports showed CpG 7909 was an effective and well-tolerated adjuvant for improving vaccine responses (Cooper et al., 2004a,b). Minor side effects were mild to moderate injection-site reactions and transient flu-like symptoms (Cooper et al., 2004a,b; Krieg, 2006). Key preclinical studies by Levy and colleagues led to development of therapeutic vaccination strategies for clinical treatment of lymphoma (Li et al., 2007; Houot and Levy, 2009; Brody et al., 2011; Goldstein et al., 2011). Combination of intratumoral CpG with cytotoxic therapy induced tumor-reactive CD8 T cells and cured primary subcutaneous and widely metastatic murine lymphomas (Li et al., 2007). Combination of intratumoral CpG and immunomodulatory T cell antibodies increased antitumor efficacy of CpG without the need for chemotherapy (Houot and Levy, 2009). A CpG-loaded tumor cell vaccine induced CD4 T cell-mediated antitumor immunity leading to regression of established murine lymphoma (Goldstein et al., 2011). A recent phase I/II clinical trial of low grade B cell lymphoma was based on the rationale that intratumoral CpG given with localized low dose radiation could be effective therapy for the primary tumor and produce immune-mediated abscopal effects (Brody et al., 2010). The *in situ* vaccination strategy with CpG ODN (PF-3512676) was well-tolerated and induced systemic antitumor responses even in patients with significant tumor burden (Brody et al., 2010). Encouraging preliminary results were also achieved in a parallel phase I/II study using a similar *in situ* vaccination strategy combined with radiation in patients with T cell lymphoma mycosis fungoides skin lesions (Kim et al., 2012).

## CONCLUSION

Treatment of mice bearing established immunogenic or non-immunogenic tumors with CpG ODN 1826 markedly enhanced response to single-dose and fractionated radiotherapy, likely through immune-mediated mechanisms. CpG ODN also induced a durable systemic immune memory response against subsequent rechallenge with tumor cells. These observations suggest CpG ODN could be used not only as an “immunosensitizer” in combination with radiotherapy but also as an adjuvant to prevent or reduce metastatic disease at sites distant from the primary irradiated tumor. These findings and others have demonstrated that CpG ODNs can be given in combination with conventional radiotherapy to improve therapeutic efficacy. Further studies are warranted to elucidate the dynamic interplay between tumor irradiation and the host immune system to facilitate translation to clinical trials. Our studies using CpG ODNs as radiation enhancing agents are being supplemented by new integrated approaches proposing a partnership between radiotherapy and immunotherapy designed to capitalize on radiation’s ability to enhance immunogenicity of the primary tumor and its microenvironment (Demaria et al., 2005; Formenti, 2010; Shiao and Coussens, 2010; Haynes and Smyth, 2012).

## Conflict of Interest Statement

The authors declare that the research was conducted in the absence of any commercial or financial relationships that could be construed as a potential conflict of interest.
